# Twenty-Five Tapeworms in One Patient

**Published:** 1910-06-04

**Authors:** 


### TWENTY-FIVE TAPEWORMS IN ONE PATIENT.

When a patient is suffering from tapeworm the question often arises as to whether he is likely to have more than one worm present at a time. In the majority of cases it is probable that the tænia will be single, but a few instances have been recorded in which three or four tapeworms have been present at once, and that even a far larger number may be present in some instances is indicated by the following case:—

The patient was a man aged thirty-one, an officer in the Army, who had been stationed at Tonkin upon two separate occasions, first for three years and again for two. During his first stay in those parts he had suffered from severe malaria and dysentery, and, moreover, he had contracted tapeworm disease. He used to pass large numbers of segments, and he stated that upon some occasions his motions consisted in nearly half their bulk of fragments and even long pieces of tapeworm, which were always very mobile. Attempts were made to cure him of his trouble, but unsuccessfully, though for some periods he was free from the passage of segments. Whenever he returned home from the tropics he either ceased to pass tapeworm segments altogether or passed very much smaller numbers of segments.

On his second return to Europe he came under observation for intermittent fever and loss of appetite. He was put upon milk diet and kept in bed, and upon the second day $1\frac{1}{2}$ drachm of oil of male fern were given in capsule form in twelve separate doses, 2 ounces of castor oil being taken half an hour after the last capsule. About two hours later he passed an enormous motion, containing a tangle of tapeworm. By careful lavage the fæcal material was separated from the worm; how much of the latter there was in total length it was impossible to tell, because it was so much tangled up, and in separating the portions they became broken; careful examination for the head was made, however, and, to the astonishment of all, it was noticed that there were several heads, so that particular search was made to determine, if possible, how many. Altogether twenty-five were discovered, and it may be that there were more.

One remarkable fact that seems worthy of particular notice is that, notwithstanding the presence of so many tapeworms in one subject, his only symptom for many years was the passage of the segments per anum; in the intervals when treatment which, though unsuccessful, had given temporary cessation in the passage of segments, he was to all intents and purposes quite well.

